# Appetite Suppression by GLP‐1 Receptor Agonists: Role of Delayed Gastric Emptying

**DOI:** 10.1002/oby.70255

**Published:** 2026-07-02

**Authors:** Mark I. Friedman, W. Scott Harmsen, Michael Camilleri

**Affiliations:** ^1^ Monell Chemical Senses Center Philadelphia Pennsylvania USA; ^2^ CENTER Program Mayo Clinic Rochester Minnesota USA

**Keywords:** appetite, food intake, gastric emptying, GLP‐1 agonists, satiation

## Abstract

**Objective:**

This study aimed to evaluate relationships between delayed gastric emptying and appetite suppression during treatment with liraglutide, a glucagon‐like peptide‐1 receptor agonist (GLP‐1RA).

**Methods:**

We conducted a secondary data analysis from a 16‐week randomized, placebo‐controlled liraglutide trial in patients with obesity. We examined correlations between solid food gastric emptying and measurements of satiation and energy intake in the entire cohorts at baseline and end of treatment and separately in placebo‐ and liraglutide‐treated patients who completed the trial. We also compared appetitive measures among liraglutide‐treated participants who displayed either normal, persistently delayed, or transiently delayed emptying during treatment.

**Results:**

In the entire cohorts at baseline and completion, gastric emptying correlated significantly with energy intake and, at baseline, with one parameter of satiation. Gastric emptying accounted for only 4%–6% of the variance in these appetitive measures. No such correlations were found in the placebo‐ or liraglutide‐treated groups alone. There were no differences in appetitive measures among the subgroups that displayed different gastric emptying patterns during liraglutide treatment.

**Conclusions:**

Delayed gastric emptying appears to play little direct role in appetite suppression induced by liraglutide. Further research should explore whether delayed emptying or other gastrointestinal effects of other GLP‐1RAs affect appetite directly or indirectly.

**Trial Registration:** ClinicalTrials.gov identifier: NCT02647944

## Abstract (highlights)

**Study Importance**

- What is already known?
  ○ GLP‐1 receptor agonists (GLP‐1RAs) reduce appetite, which may be due in part to delayed gastric emptying.
- What does this study add?
  ○ While there may be overall modest correlations between gastric emptying and measures of satiation and energy intake, there is little evidence for such a relationship in liraglutide‐treated patients.
- How might these results change the direction of research or the focus of clinical practice?
  ○ Identifying the precise mechanism(s) for reduced appetite with GLP‐1RAs requires further research, including into indirect effects of delayed gastric emptying on endogenous appetite‐related hormones and fuel metabolism.

## 1 Introduction

Glucagon‐like peptide‐1 receptor agonists (GLP‐1RAs) appear to induce weight loss primarily by reducing food intake [1]. GLP‐1RAs are thought to suppress appetite by acting directly on hypothalamic and brainstem neuronal circuits controlling hunger, satiety, and eating behavior, but they also can delay gastric emptying (GE), which is often considered a contributing factor associated with reduced food intake [1]. The relationship between appetite suppression and delayed GE effects of GLP‐1RA treatment has not been elucidated. Many prior studies have frequently used a paracetamol/acetaminophen absorption test, which is primarily indicative of the early 1 to 2‐h liquid phase of GE, whereas assessments of appetite or test meal intake are typically made using solid food over or after longer (e.g., 4–5 h) intervals [2, 3]. Measurement of the slower solid phase of food emptying using nuclear scintigraphy [4, 5] shows that GLP‐1RAs delay solid food emptying with increased retention of a standardized meal extending beyond 3 h after ingestion [6, 7]. Therefore, the relationship between appetite and GE would be best explored in the context of solid emptying.

In the only available study that has examined prospectively the effects of GLP‐1RA treatment on both appetitive measures and GE of solid food [6], patients treated with liraglutide evidenced delayed GE, increased satiety, and decreased test meal energy intake. Here, we report the results of a secondary analysis of these data to specifically examine the relationship between GE and reduced appetite during GLP‐1RA treatment.

## 2 Methods

### 2.1 Study Design and Participants

We analyzed data from a previously published [6] randomized, parallel‐group, placebo‐controlled, 16‐week trial of once‐daily liraglutide (escalated to 3 mg subcutaneously) in 136 otherwise healthy adults (18–65 years of age) with obesity (BMI > 30 kg/m^2^) conducted at the Mayo Clinic in Rochester, Minnesota. The study was approved by the Mayo Clinic Institutional Review Board (IRB No. 15–001783) and registered with ClinicalTrials.gov (NCT02647944). All participants provided written informed consent.

### 2.2 Measurements

Methods to measure GE of solids [8], satiation [9], and food intake [10] have been detailed previously. Briefly, solid food emptying was measured by scintigraphy after ingestion of a 320‐kcal ^99m^Tc‐radiolabeled egg, solid–liquid meal. The rate of emptying was calculated as the proportion of the meal emptied at 2 h and as the time taken for half the radiolabeled meal to empty from the stomach (T_1/2_). Patients were selected based on having normal GE T_1/2_ (< 175 min) at baseline. With respect to appetitive measures, satiation was assessed by measuring volume to fullness (VTF) and maximum tolerated volume (MTV) during ingestion of Ensure (1 kcal/mL) at a constant rate of 30 mL/min. Energy intake (kcal) was determined by measuring ad libitum consumption of a buffet test meal consisting of standard foods of known nutrient composition.

### 2.3 Statistical Analyses

Descriptive statistics are reported as numbers (percentages) for discrete variables or means (SD) for continuous variables.

To assess the relationship between GE and appetite, we performed univariate linear regression between both GE measures and each of the three appetitive measures at baseline in all 136 participants, in all 116 participants who completed the study at 16 weeks, and, separately, in those who had been treated with either placebo or liraglutide (*N* = 60 and 56, respectively). Multivariate regression assessed whether at 16 weeks the relationships between GE at 2 h or GE T_1/2_ and kcal intake during the test meal were significantly different in the placebo‐ and liraglutide‐treated patients.

To more directly test the relationship, we examined appetitive measures taken at the end of the trial among three previously identified [11] subgroups of the 59 liraglutide‐treated patients who completed the study. These groups of patients showed either (i) normal rates of emptying throughout the trial (*N* = 24); (ii) a persistent delay in emptying throughout the trial (*N* = 16); or (iii) tachyphylaxis with delayed emptying at 5 weeks, reverting to normal by 16 weeks (transient delay; *N* = 19). Univariate regression was used to assess correlations between GE and kcal intake in the different subgroups. Comparisons of appetitive measures among groups were made using analysis of variance, analysis of covariance with baseline values as covariates, and the Kruskal Wallis test for ranked values.

The alpha level was set at 0.05 for statistical significance. All analyses were completed using SAS version 9.4.

## 3 Results

### 3.1 Baseline

**FIGURE 1:**
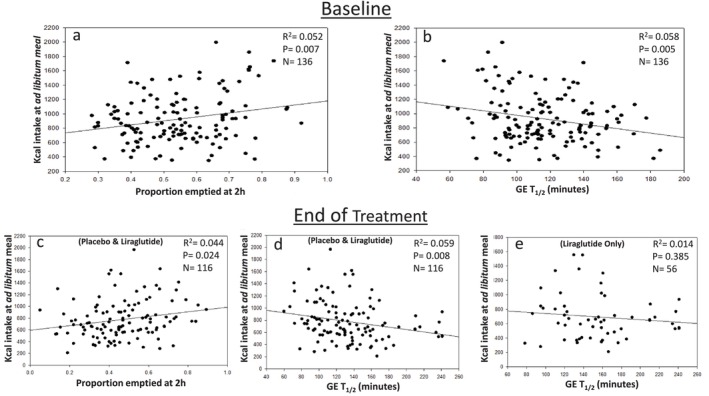
Correlations between gastric emptying and energy (kcal) intake of an ad libitum buffet test meal before and after patients were randomly assigned to placebo or liraglutide treatment. Results are shown for (a,b) all patients at baseline and (c,d) at the end of the 16‐week trial for both placebo‐ and liraglutide‐treated patients or (e) only those treated with liraglutide. Gastric emptying was measured by scintigraphy as the proportion of the meal emptied at 2 h (panels a, c) and as the time taken for half the radiolabeled meal to empty from the stomach (GE T_1/2_; panels b, d, e).

At baseline, GE at 2 h and GE T_1/2_ were significantly correlated with kcal intake at the ad libitum meal, specifically 55.67‐kcal lower intake per 10% lower GE at 2 h (*p* = 0.0074) and 62.66‐kcal lower intake per 20‐min slower GE T_1/2_ (*p* = 0.0049). These significant effects on GE accounted for 5%–6% of the variance (based on *R* ^2^) in kcal intakes (Figure 1a,b). Neither measure of GE was significantly associated with either VTF or MTV.

### 3.2 End of Treatment

Considering all patients who completed the 16‐week trial, GE at 2 h and GE T_1/2_ were significantly associated with kcal intake (Figure 1c,d), specifically 39.33‐kcal lower intake per 10% decreased emptying at 2 h (*p* = 0.024) and 39.55‐kcal lower intake per 20‐min slower GE T_1/2_ (*p* = 0.008). GE T_1/2_ was significantly (*p* = 0.026) associated with a decrease in MTV (33.99‐lower kcal per 20‐min slower T_1/2_), but not with VTF (7.95‐lower kcal per 20‐min slower T_1/2_; *p* = 0.45). These significant effects of GE accounted for 4% and 6% of variance (based on *R* ^2^) in the appetitive measures, respectively.

There were no significant correlations of either GE parameter with any appetitive measure at the end of treatment in either the placebo or, as shown in Figure 1e for kcal intake, the liraglutide groups. For example, GE T_1/2_ was not significantly associated with test meal intakes in either the placebo or liraglutide groups (respectively, 48.22 and 17.27 lower kcal per 20 min slower; *p* = 0.14 and 0.39). These relationships between GE and kcal intake also did not differ significantly between the two groups (*p* = 0.38 for interaction).

**FIGURE 2:**
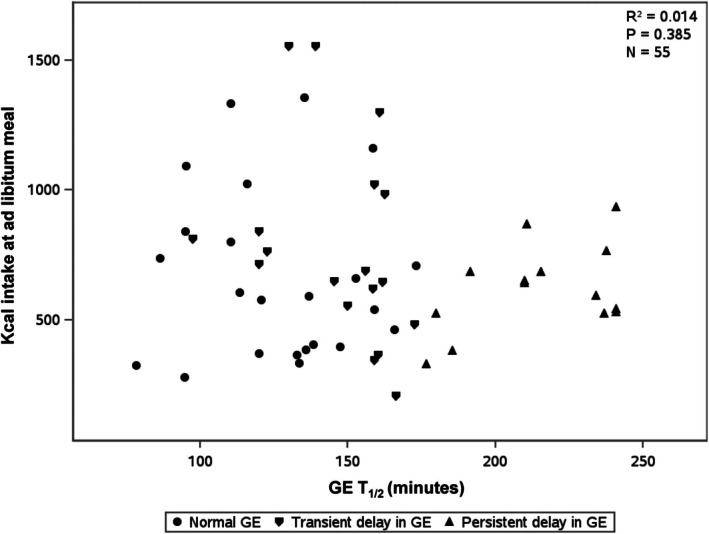
Relationships between gastric emptying (GE T_1/2_) and energy (kcal) intake of an ad libitum buffet test meal in liraglutide‐treated patients at end of trial who displayed either normal GE (no change in emptying from baseline); persistent delay in GE (delayed emptying throughout the trial); or transient delay in GE (delayed emptying at 5 weeks, which then reverted to normal by 16 weeks). Univariate regression analysis showed no statistically significant correlations between gastric emptying and kcal intake in the subgroups, which are reflected in the dispersion of individual data points for participants in the three groups.

Correlations between GE T_1/2_ and kcal intake in the test meal did not differ significantly in the subgroups with different GE profiles (Figure 2). *R* ^2^ values for the subgroups showing consistently normal, consistently delayed, and transiently delayed GE were, respectively, 0.0034, 0.1935, and 0.098 (*p* = 0.791, 0.116, and 0.206).

**FIGURE 3:**
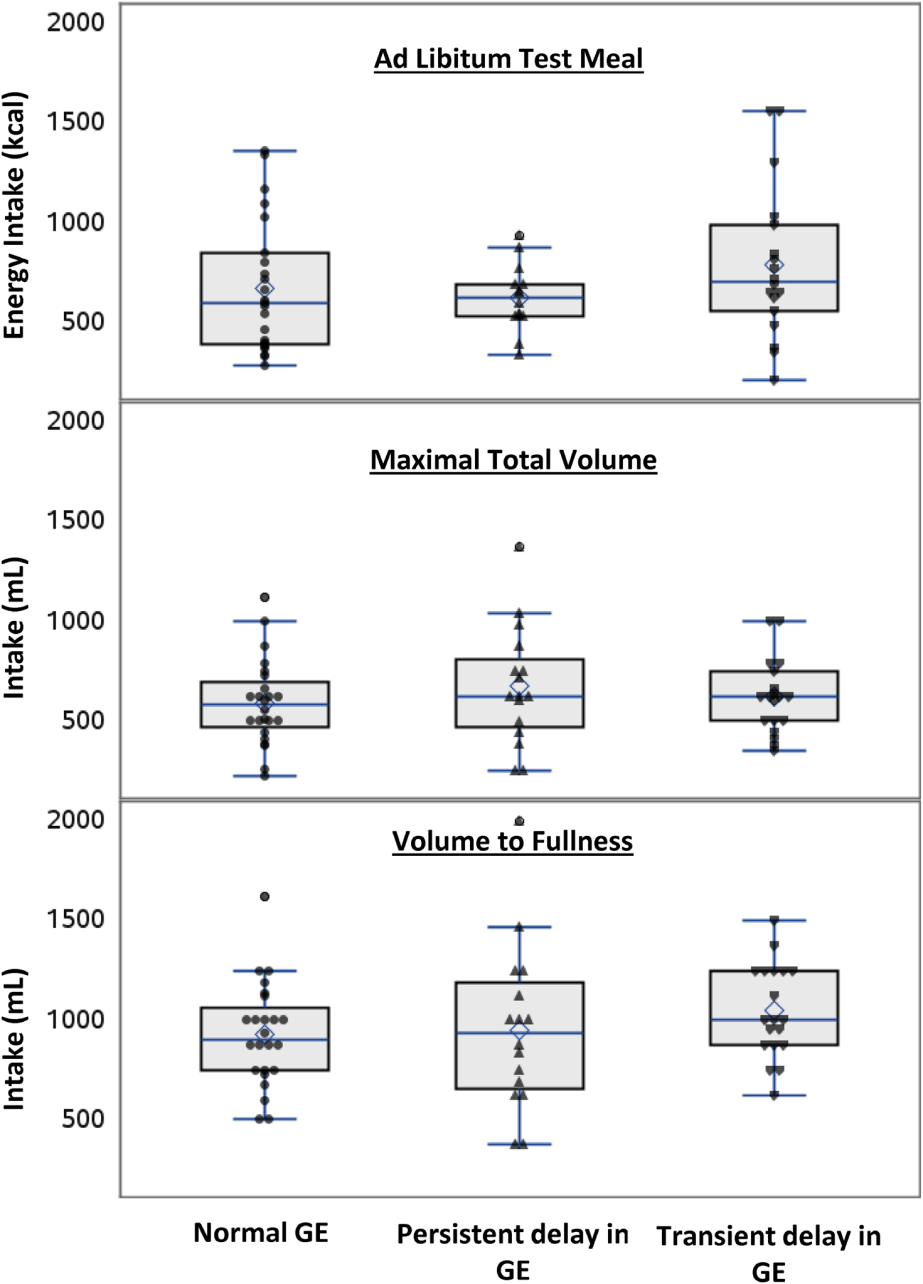
Appetitive measures in liraglutide‐treated patients who showed different profiles of gastric emptying (GE) throughout the 16‐week trial. Normal GE (no change in emptying from baseline *N* = 24); persistent delay in GE (delayed emptying throughout the trial *N* = 16); and transient delay in GE (delayed emptying at 5 weeks, which then reverted to normal by 16 weeks *N* = 19). Ad libitum test meal = intake (kcal) of a buffet meal; maximum total volume and volume to fullness = amount (mL) consumed during ingestion of Ensure at a constant rate of 30 mL/min. Elements are median and mean (line and diamond, respectively, within the interquartile range box); whiskers show the minimum and maximum range of individual data points. There were no statistically significant differences in appetitive measures among the groups as assessed by analysis of variance, analysis of covariance, or the Kruskal–Wallis test. [Color figure can be viewed at wileyonlinelibrary.com]

Neither ad libitum meal kcal intake, MTV, nor VTF differed significantly among the subgroups, whether by analysis of variance or covariance (*p* > 0.32 and 0.48, respectively) or a Kruskal Wallis test (*p* > 0.26) for all three assessments (Figure 3).

## 4 Discussion

These analyses suggest that, under the conditions of this randomized trial, there were modest relationships between GE and appetitive measures of satiation and energy intake at baseline and at end of treatment in the entire patient cohorts. Consistent with previous findings [12], there were significant, but relatively low, correlation coefficients between GE and satiation—specifically, MTV—and test meal energy intake in all study participants. However, no such relationships were found within either the placebo‐ or liraglutide‐treated groups. The subgroup analyses also suggest a lack of relationship among patients with the three distinctly different GE profiles and appetitive effects of GLP‐1RA treatment at the end of the 16‐week trial.

There are limitations in this secondary analysis. For example, smaller sample sizes of liraglutide‐treated subgroups with different GE profiles may have underpowered the analyses. Lower potency of liraglutide relative to more recently developed GLP‐1RAs may have diminished the relationship between GE and appetitive measures. It is also conceivable that other measures of appetite might be better associated with delayed GE. GLP‐1RAs generally reduce self‐reported, subjective measures of appetite after a standardized meal as measured using visual analogue scales, although differences among ratings for individual descriptors (e.g., hunger, satiety, fullness, prospective intake) or overall scores are often not statistically significant [2]. On the other hand, consistent with present findings, self‐reported appetite is reduced in patients being treated with GLP‐1RAs after an overnight fast [2, 13] when any effect on GE or gastric volume would be expected to be minimal [6].

In the liraglutide group alone, there were no significant relationships of either GE parameter with any appetitive measure (Figures 1e, 2, 3). Despite this lack of a relationship between GE and appetitive measures, the observation that GE nevertheless accounted for a significant proportion of weight loss in liraglutide‐treated patients [6] raises the possibility that gastrointestinal effects of liraglutide treatment may affect weight loss independent of its effect on food intake. In this regard, based on infusion studies with GLP‐1, it has been suggested [14] that slow GE induced by GLP‐1RA treatment may reduce postprandial glycemia and insulinemia, which would be expected to facilitate loss of body fat. The findings also suggest that, rather than resulting directly from the slowing of GE, suppression of appetite by liraglutide may be related to other actions of GLP‐1RAs, including effects on neuronal circuits controlling food intake, on mechanisms eliciting nausea, particularly in the early phases of treatment [15], or by altering the disposition and utilization of metabolic fuels [16].

## 5 Conclusion

The effects of liraglutide on appetite appear to be only marginally related to delayed GE. Additional research is needed to determine to what extent delayed GE plays a role in appetite suppression and reduction of food intake induced by other GLP‐1RAs or by newer incretin co‐agonists.

## Funding

This work was supported by National Institutes of Health, RO1 DK67071 and RO1 DK142606.

## Conflicts of Interest

The authors declare no conflicts of interest. M.C. serves as an advisor to Lilly with compensation to Mayo Clinic, not to himself personally.

## Data Availability

The data that support the findings of this study are available from Dr. Camilleri upon reasonable request.
